# First Case of Human Ocular Dirofilariasis in the Aosta Valley Region: Clinical Management and Morphological-Molecular Confirmation

**DOI:** 10.3390/pathogens14050423

**Published:** 2025-04-28

**Authors:** Erik Mus, Annalisa Viani, Lorenzo Domenis, Fabio Maradei, Antonio Valastro, Gianluca Marucci, Claudio Giuseppe Giacomazzi, Silvia Carla Maria Magnani, Roberto Imparato, Annie Cometto, Adriano Casulli, Riccardo Orusa, Luca Ventre

**Affiliations:** 1 Department of Ophthalmology, Beauregard Hospital, Azienda USL della Valle d’Aosta, Via L. Vaccari 5, 11100 Aosta, Italy; lventre@ausl.vda.it (L.V.);; 2 S.C. Animal Health, Azienda USL della Valle d’Aosta, Località Amerique 7/l, 11020 Quart, Italy; aviani@ausl.vda.it; 3 Experimental Zooprofilactic Institute of Piedmont, Liguria, and Aosta Valley National Reference Centre for Wildlife Diseases (CeRMAS), Loc. Amerique 7/G, 11020 Quart, Italyriccardo.orusa@izsplv.it (R.O.); 4 European Union Reference Laboratory for Parasites (EURL-P), Unit of Foodborne and Neglected Parasitic Diseases, Istituto Superiore di Sanità, Viale Regina Elena 299, 00161 Rome, Italy; 5 Microbiology Unit, U. Parini Hospital, Azienda USL della Valle d’Aosta, 11100 Aosta, Italy; 6 Infectious Diseases Unit, U. Parini Hospital, Azienda USL della Valle d’Aosta, 11100 Aosta, Italy

**Keywords:** human ocular dirofilariasis, *Dirofilaria repens*, filarioid nematodes, public health, zoonoses, emerging diseases, One Health

## Abstract

**Purpose:** Dirofilariasis is a zoonotic infectious disease caused by a species belonging to the *Dirofilaria* genus. Human dirofilariasis cases have increased in Europe in the last few decades. Dogs and wild canids represent the definitive hosts and principal reservoirs of *Dirofilaria repens,* while mosquito species are biological vectors. Humans act as accidental hosts, and clinical manifestations depend on the location of the worm in the organs or tissues. We described the first case of ocular dirofilariasis in the Aosta Valley region (Italy). **Case description:** a 62-year-old Italian woman complained of recurrent ocular redness, pain and discomfort, accompanied by itching and foreign body sensation in the right eye. The slit lamp biomicroscopic examination revealed conjunctival congestion on the temporal region of bulbar conjunctiva, and a long whitish vermiform mobile mass was detected under the conjunctiva. The anterior chamber showed no flare or cells in either eye, and the dilated fundus examination was normal. The worm was immediately surgically removed to prevent further migration, and was diagnosed morphologically and molecularly as *D. repens*. Following surgical removal, the symptoms resolved completely and rapidly, with no recurrence of ocular symptoms recorded during 12-month follow-up visits. **Conclusions:** Ocular dirofilariasis can lead to misdiagnosis due to its rare ocular manifestations, and it is considered an emergent zoonosis in European countries. Accurate diagnosis and control of ocular dirofilariasis by *D. repens* require a multidisciplinary approach under the One Health framework to effectively address this emergent zoonosis.

## 1. Introduction

Dirofilariasis is a zoonotic infectious disease caused by filarioid nematodes of the genus *Dirofilaria* (Spirurida, Onchocercidae) [1]. Approximately 40 species have been identified, and the two most common infecting humans are *Dirofilaria repens,* associated with subcutaneous and ocular pathologies, and *Dirofilaria immitis,* responsible for pulmonary dirofilariasis [2,3]. Dogs and wild canids represent the definitive hosts and principal reservoirs of *D. repens,* while felids are rarely positive for circulating microfilariae. The presence of autochthonous *D. repens* infections has been demonstrated in several European countries [4]. Nevertheless, the highest incidence of human cases has been found in Italy, Greece, and France, and in the last twenty years, also in Ukraine, the Russian Federation, and Belarus [5].

Regarding wildlife, the parasite can also infect wild carnivores, such as red foxes and wolves [6,7]. In some anthropized environments, contact between wildlife and domestic animals could represent the initial step in transmission to humans. The raccoon dog (for *D. immitis*) and the badger (for *D. repens*) have been reported as new hosts in Europe. Sparse cases of *Dirofilaria* infections in raccoon dogs exist in the literature, reported only in Japan [8,9,10,11]. A recent study conducted in the Russian Federation also identified badgers as new hosts of *D. repens* in Europe [12].

Because of the biological characteristics of the parasite, mosquito species belonging to different genera (i.e., *Anopheles*, *Aedes*, *Culex*, and *Coquillettidia*) may be involved in the life cycle of the parasite as competent vectors [5]. The involvement of vectors is epidemiologically relevant. It is worth noting that climate change has widely influenced mosquito habitats, promoting the colonization by mosquitoes of new ecological areas worldwide. The vectorial capacity of a mosquito species for a specific pathogen is influenced by several factors, such as population density, seasonality, host availability, etc. [5]. For the transmission of *D. repens* L3 larvae to a canine (or other vertebrate) host, an infected mosquito must survive at least until the end of the extrinsic incubation period, during which the highly motile L3 will have reached proboscis. Furthermore, the mosquito species must be endemic to areas where dogs are present, and they must have a biting preference for canines.

It is evident that environmental conditions must be closely monitored to provide information about future spreads of infectious diseases. The development of new technologies provides interesting tools for the evaluation of environmental variables that may influence the life cycle of various pathogens, directly or indirectly. In this context, the use of Earth observation (EO) data allows researchers to create suitability maps for the detection and assessment of different diseases, with particular regard to zoonotic diseases. About 75% of the emerging human diseases recognized in recent decades have a zoonotic origin. Furthermore, 70% of zoonoses are transmitted by wildlife, and this number has unexpectedly increased in recent years. Moreover, pet ownership has risen significantly: from companionship to emotional support, pets are a vital part of their owners’ lives. This situation increases the probability of contact between humans and animals, and facilitates the transmission of pathogens from one host to another.

Nowadays, dirofilariasis is endemic in many countries in the Old World, mainly because of poor hygienic conditions and inadequate health education. Humans are accidental hosts [2], and the parasite usually does not reach the adult stage, remaining confined to an immature form. This may result in the development of subcutaneous nodules (larva migrans syndrome). The worm is often localized in the ocular region and, occasionally, in other organs, such as the lungs.

Human dirofilariasis is an emerging disease and a public health concern in many parts of the world, including Europe, Asia Minor, Central Asia, and Sri Lanka [2,3,13,14].

Concerning Europe, *D. repens* is an endemic nematode in the southern countries (Italy, Spain, France, and Greece), and particularly in the Mediterranean basin [1,2,15]. In Italy, *D. repens* is endemic throughout the peninsula, including the major islands (Sicily and Sardinia), with a prevalence ranging between 1.5 and 12%. Moreover, the literature reports co-infections in dogs with filarioids, such as *Acanthocheilonema reconditum* and *D. immitis* [7,16,17]. *D. repens* has also been detected in *Culex pipiens* mosquitoes in the northeastern part of the country, with an infection rate ranging between 0.23 and 0.71% [18]. Italy reports some of the highest numbers of human cases, with case series involving up to 60 patients [2,19,20]. These human cases are spatially correlated with areas where infections in dogs are endemic [21,22].

The aim of the present work was to describe the first subconjunctival case of *D. repens* infection that occurred in the Aosta Valley region (Northwest Italy) and the identification process of the nematode, pointing out the importance of collaboration between different institutions and public health sectors in Italy.

## 2. Materials and Methods

### 2.1. Case Description

A 62-year-old woman presented to the accident and emergency department for ophthalmology at Beauregard Hospital (Aosta Valley region, Azienda USL della Valle d’Aosta, Italy), complaining of recurrent ocular redness, pain and discomfort associated with itching, and foreign body sensation in the right eye. The patient reported experiencing an edema of the right eyelid three months earlier, which caused her significant difficulty in opening the eye. She was treated with antibiotics and corticosteroid eye drops, with subjective improvement. Her general medical history reported nothing relevant. She had no history of international travel and had no prior episodes of similar symptoms. The patient lives in the countryside (450 m above sea level) and keeps various animals, including dogs and cats. Lastly, the patient did not report any insect bites that were possibly painful or allergic to the skin before the onset of symptoms.

**Figure 1:**
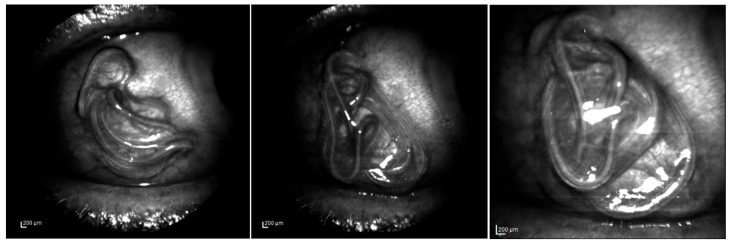
Some frames from the video recorded in infrared reflectance (IR) mode, using the Spectralis HRA (Heidelberg engineering), during the ocular examination prior to the *Dirofilaria repens* worm removal. The images were acquired by a 55° lens in the first and second frames and a 35° lens in the third one.

Slit lamp biomicroscopic examination of the right eye revealed conjunctival congestion in the temporal region of the bulbar conjunctiva, and the presence of a long whitish vermiform mobile mass under the conjunctiva in the temporal part of the right eye (Figure 1). The anterior chamber of both eyes showed no flare or cells, and the dilated fundus examination was normal. The remainder of the ocular examination did not reveal any abnormalities, and visual acuity was 20/20 in both eyes.

A combination of 10% Phenylephrine and Oxybuprocain eye drops was instilled in the conjunctival sac to reduce the worm’s motility, although this approach was not well described in the literature.

**Figure 2:**
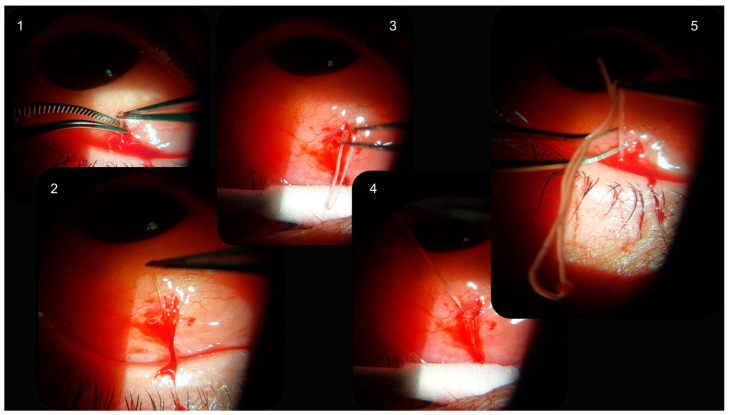
Selected frames from the video recorded with the slit lamp during the *Dirofilaria repens* worm removal.

To prevent further migration of the worm, a surgical procedure under local anesthesia was performed immediately. Following a subconjunctival injection of 1 mL lidocaine (20 mg/mL), the conjunctiva was incised using Westcott scissors to expose the worm and a live, thin, segmented, whitish, worm was entirely excised from the sub-Tenon’s space (Figure 2).

On the same day as the worm removal, the patient was referred to the infectiologist for further examination to rule out the presence of additional parasites elsewhere in the body.

Following the surgical removal, the patient’s symptoms resolved rapidly and completely, with no recurrence of the ocular symptomatology recorded at the 1, 3, 6 and 12-month follow-up visits. The excision site healed uneventfully within one month.

**Figure 3:**
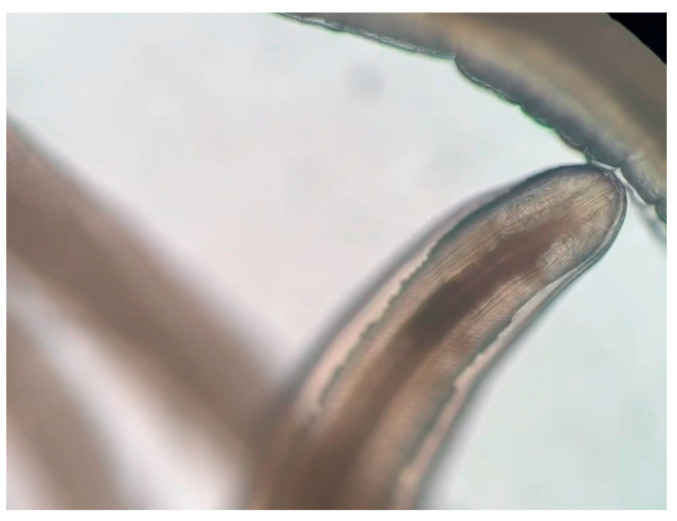
Photo of the just-removed worm from the patient’s right eye, visualized using 40× magnification with an optical microscope, Nikon Eclipse 50i (Nikon, Tokyo, Japan).

The removed parasite was stored in a sterile test tube filled with physiological saline, and sent to the microbiology unit of Umberto Parini Hospital in Aosta (Figure 3). Subsequently, the sample was forwarded to the Experimental Zooprophylactic Institute of Piedmont, Liguria and the Aosta Valley in Quart (AO) for species identification.

At the Institute, the worm was evaluated by visual examination under an Olympus BZ40 stereomicroscope to assess its macroscopic features and measure its length using a ruler and DP Soft Olympus imaging software (version 3.1). The worm was then sectioned in three pieces: the two extremities were subjected to histological examination by a routine method (fixed in 90% alcohol, paraffin embedded, cut into slices 3–4 µm thick, and stained with hematoxylin–eosin), and examined at different magnifications under an optical microscope. The middle section was sent to the European Union Reference Laboratory for Parasites (EURL-P; https://www.iss.it/en/eurlp-chi-siamo, accessed on 23 April 2025), Unit of Foodborne and Neglected Parasitic Diseases of the Italian National Institute of Health (Istituto Superiore di Sanità, ISS) in Rome for molecular identification.

### 2.2. Molecular Identification

DNA purification was carried out using the DNA IQ System and Tissue and Hair Extraction kit (Promega, Madison, WI, USA) according to the manufacturer’s protocol. For molecular identification, specific PCR primers targeting the 18S rRNA gene [23], 12S gene [24], and cytochrome c oxidase subunit I (CO1) [25] were used according to the authors’ protocols. PCR products were purified using the QIAquick PCR Purification Kit (Qiagen, Hilden, Germany) and sent to Eurofins Genomics (Ebersberg, Germany) for standard Sanger sequencing. The sequences were analyzed by the CLC Genomic Workbench (Qiagen, Hilden, Germany) and compared with the GenBank database by the Basic Local Alignment Search Tool (BLAST +2.16.0) [23,24,25].

## 3. Results

### 3.1. Morphology

**Figure 4:**
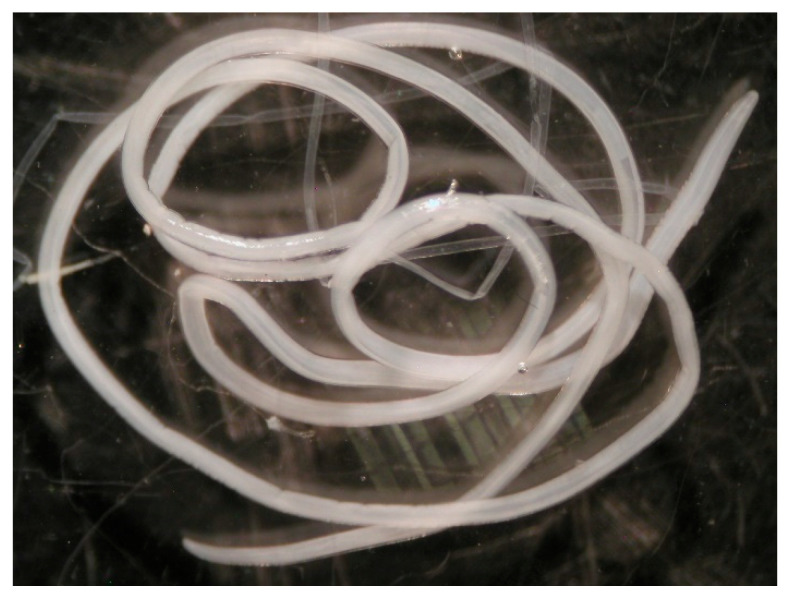
*Dirofilaria repens* specimen removed from the patient.

**Figure 5:**
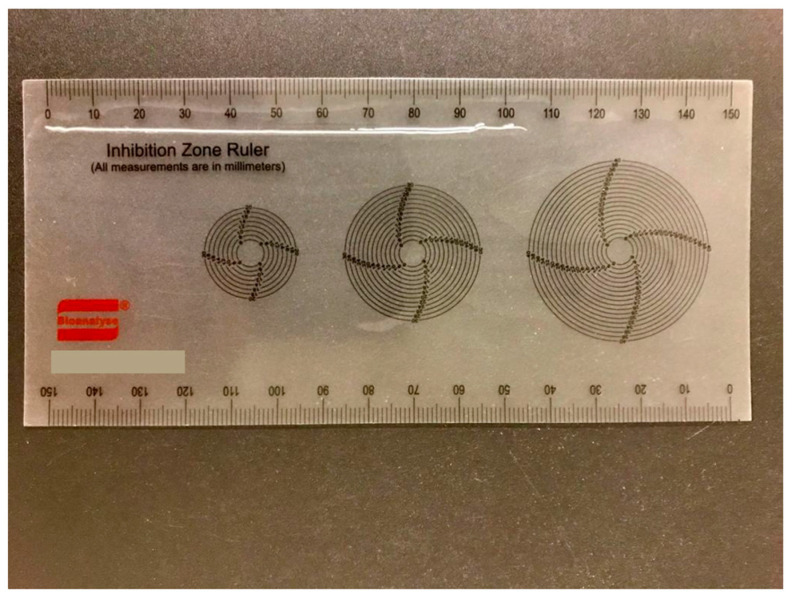
Measurement of *Dirofilaria repens*.

**Figure 6:**
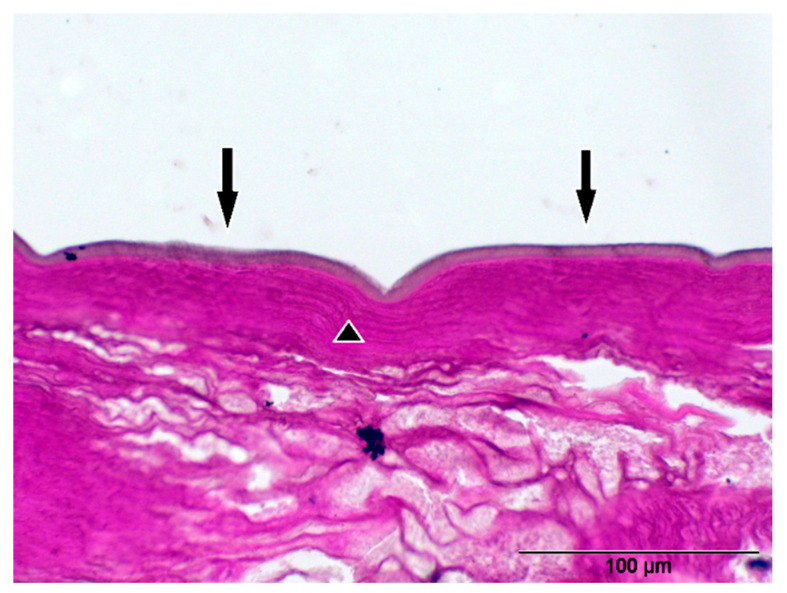
*Dirofilaria repens* (40× magnification and stained with H&E) with corrugated cuticle (arrow) and striated muscle layer (triangle) in longitudinal section.

**Figure 7:**
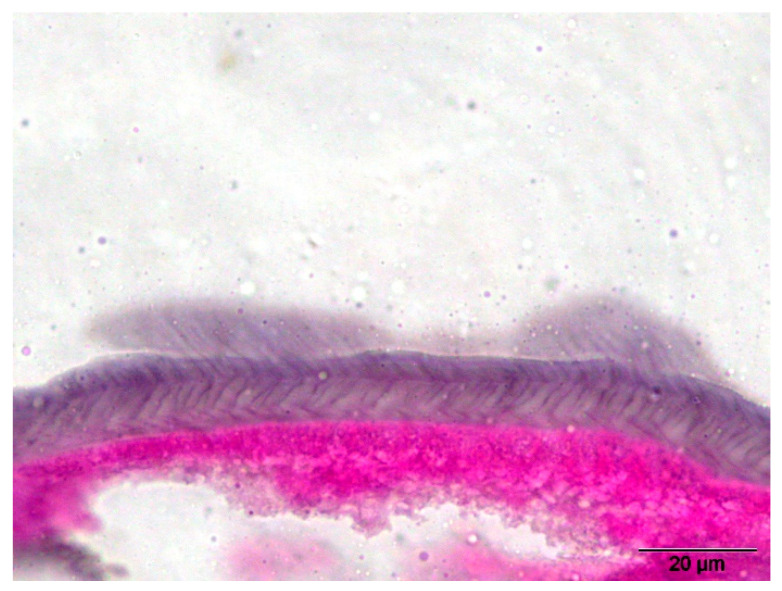
*Dirofilaria repens*—multilayered cuticle with transverse striations (100× magnification with H&E staining).

Upon visual examination, the worm appeared filiform, cylindrical, and whitish, with rounded ends (Figure 4). The total length was 10.5 cm (Figure 5). Microscopically, the extremities of the nematode showed a typical corrugated, thick multilayered cuticle (Figure 6) with transverse striations (Figure 7) and a muscular striated layer beneath. By comparing these features with bibliographic references, a diagnosis of *Dirofilaria* spp. was suggested [26,27,28,29].

### 3.2. Serological and Stool Analysis

General blood tests were normal. Three stool samples, submitted to the microbiology unit of the Umberto Parini Hospital (Aosta Valley region) for parasitological examination, yielded negative results. Finally, a serological sample was submitted to the microbiology unit of “Amedeo di Savoia” hospital in Turin (Piemonte region) to investigate Toxocara spp. Antibodies, and surprisingly, IgG results were positive when using a blot method (Toxocara WB IgG, Ldbio Diagnostics, Lyon, France). Since IgG blots are used as a confirmatory test, the microbiology unit concluded that this result should not be considered a cross-reaction.

### 3.3. Molecular Identification

The PCR, using specific primers targeting variable regions inside the 18S, 12S, and CO1 genes, resulted in DNA fragments of 904 bp, 464 bp, and 671 bp, respectively. All three sequences obtained were analyzed by the BLAST algorithm, which showed 100% similarity with the homologous sequences of *Dirofilaria repens* deposited in GenBank, allowing for the unequivocal identification of the parasite.

## 4. Discussion

This case represents an example of ocular dirofilariasis, a rare condition caused by the parasite *Dirofilaria repens*. Concerning the serological results, the section of microbiology suggested that the IgG positivity to *Toxocara* was not correlated with ocular dirofilariasis, as it could be a sign of previous asymptomatic exposure to this parasite. However, this positivity could have led to the incorrect identification of the worm. 

Our report highlights the diagnostic challenges of recognizing rare zoonotic infections such as ocular dirofilariasis. The reasons for this are associated with the limited use of routine tests and the non-pathognomonic symptoms of dirofilariasis, which can be easily confused with other conditions [30].

The diagnosis of ocular dirofilariasis is based on the microscopic identification of the worm extracted from the lesion or from the examination of a histological section of a biopsy [1]. As in our case, serological diagnosis typically does not provide sufficient interspecific discrimination [1]. Molecular techniques are a very useful complementary tool, especially where the parasite’s morphology is altered by the host’s immune response or where the parasite’s microhabitat leads to confusion [1].

In the case of dirofilariasis, ophthalmic involvement may occur in various forms, including orbital (lacrimal gland and sac) [31], periorbital (lids) [32], subconjunctival [1,33,34], sub-Tenon [35], and intraocular (anterior or vitreous chamber) [36,37] associations.

The surgical removal of the worm is the treatment of choice, and it is usually curative, as pharmacological treatments are usually unnecessary and ineffective [1,38].

Ocular dirofilariasis can be considered an emerging zoonosis that requires a multidisciplinary approach. It is worth noting that collaboration between different scientific sectors is key to the identification, management, and resolution of several diseases occurring at the human/animal interface. These considerations have acquired increased importance in recent years, particularly with the affirmation of the One Health concept [39].

Dirofilariasis prevention is fundamental in areas where these mosquitoes might carry the parasite, and involves control measures such as disinfestation, the use of repellents and protective clothing, limiting outdoor exposure, and administering antiparasitic treatments to pets to prevent mosquito bites.

This last point helps to break the life cycle of the parasite and reduces the risk of transmission to humans. For this reason, pet owners should be made aware and should consult their veterinarians about regular preventive treatments for heartworm (dirofilariasis) [5].

## 5. Conclusions

The present work reports the first detection of a *D. repens* ocular infection in the Aosta Valley region (Italy). The case described represents an important example of a multidisciplinary collaboration between different public health institutions. More than ever, adopting a One Health approach is essential to treat infectious diseases and create partnerships among various scientific disciplines, such as human medicine, veterinary medicine, biology, and public health.

Unfortunately, due to the lack of an official national register, and because of the limited references in the literature regarding reports of this parasitosis in Italy, a retrospective epidemiological analysis of the cases of human ocular dirofilariasis in our country could not be conducted. It is fundamental to encourage proper case reporting at the hospital level, including molecular characterization of the parasitic species involved.

Therefore, establishing permanent partnerships between universities and research centers of excellence is essential. Such collaborations could facilitate the exploration of new research directions and the documentation of new interesting epidemiological situations, contributing to improving the knowledge in specific fields.

## Data Availability

The original contributions presented in this study are included in the article. Further inquiries can be directed to the corresponding author.
